# Fourth case of tegumentary leishmaniasis in Brazil by *Leishmania major* ‒ is it possible for new species to be introduced in Brazil through immigration?^☆^

**DOI:** 10.1016/j.abd.2022.07.004

**Published:** 2023-03-13

**Authors:** Cindy Tiemi Matsumoto, Milvia Maria Simões e Silva Enokihara, Marília Marufuji Ogawa, Samira Yarak

**Affiliations:** Department of Dermatology, Escola Paulista de Medicina, Universidade Federal de São Paulo, São Paulo, SP, Brazil

Dear Editor,

Tegumentary leishmaniasis (TL) in Brazil is the New World tegumentary leishmaniasis (NWTL), with distinct protozoan species, vectors, reservoirs and presentation when compared to the Old World tegumentary leishmaniasis (OWTL), which occurs in European, African and Middle Eastern countries. The authors report the fourth case of TL in Brazil caused by an Old World species, which is not native to Latin America, and discuss the introduction of this species in the country.

## Case report

**Figure 1:**
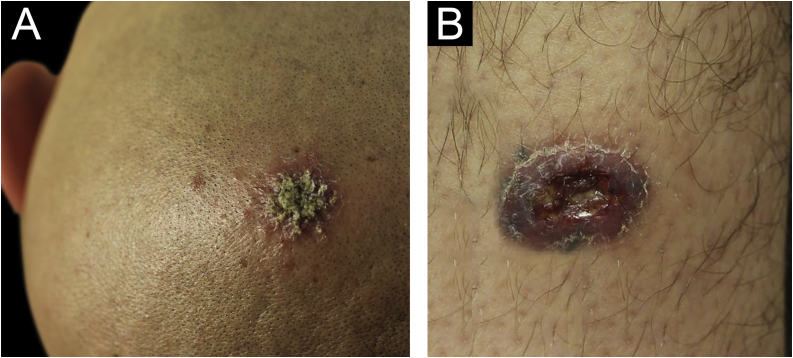
(A) Erythematous plaque covered by a meliceric crust on the scalp. (B) Ulceration with clear bottom and framed edges, above the right medial malleolus.

**Figure 2:**
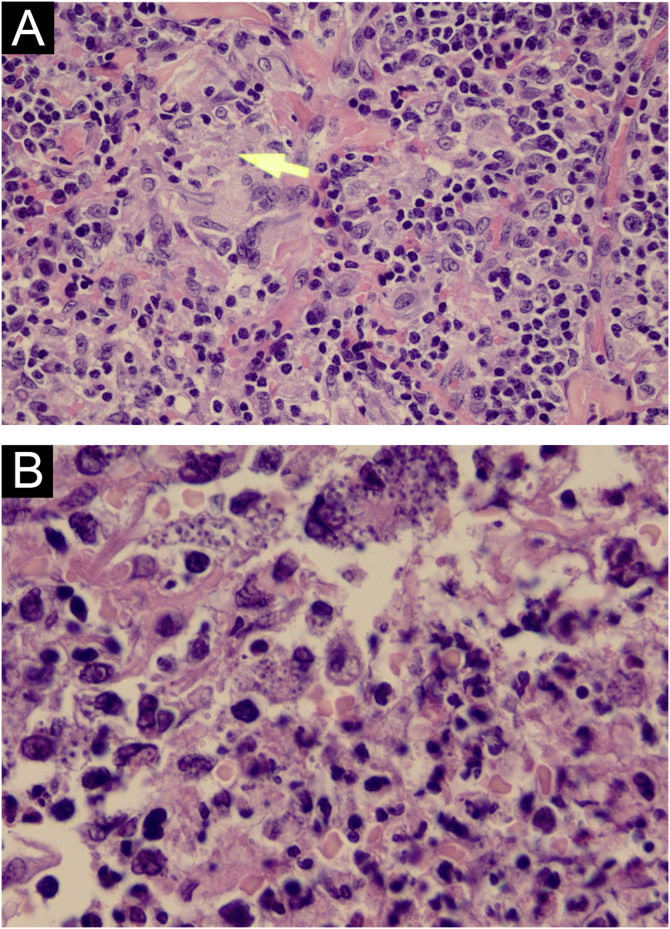
(A) Amastigote parasites inside macrophages (yellow arrow; Hematoxylin & eosin, ×400). (B) Amastigote parasites inside macrophages (Hematoxylin & eosin, ×1000).

**Figure 3:**
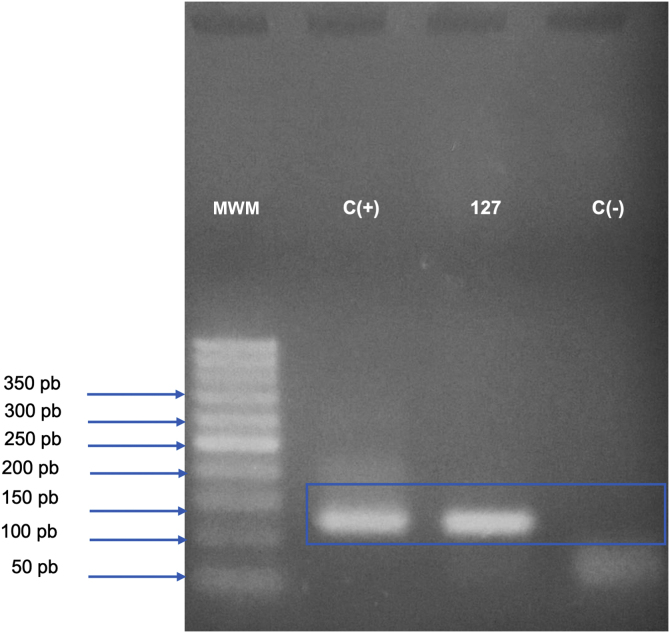
Analysis of the kinetoplast minicircle DNA fragment (mitochondrial DNA or kDNA) of *Leishmania spp*., amplified by conventional Polymerase Chain Reaction (PCR) and detected by electrophoresis in 2.5% agarose gel. The bands observed on the gel represent the PCR product (amplicon) of 116‒120 base pairs, generated from the amplification with kDNA-F and kDNA-R primers; the gel was stained with Safe Dye (Cellco Biotech do Brasil). MWM, 50 bp Molecular Weight Marker (Cellco Biotech do Brasil); C(+), Positive control (DNA extracted from culture ‒ *Leishmania amazonensis* – 40 ng/L); 127, DNA extracted from a skin biopsy specimen from the right medial malleolus region, fixed in formalin and paraffin-embedded; C(-), Negative control (sterile water).

**Figure 4:**
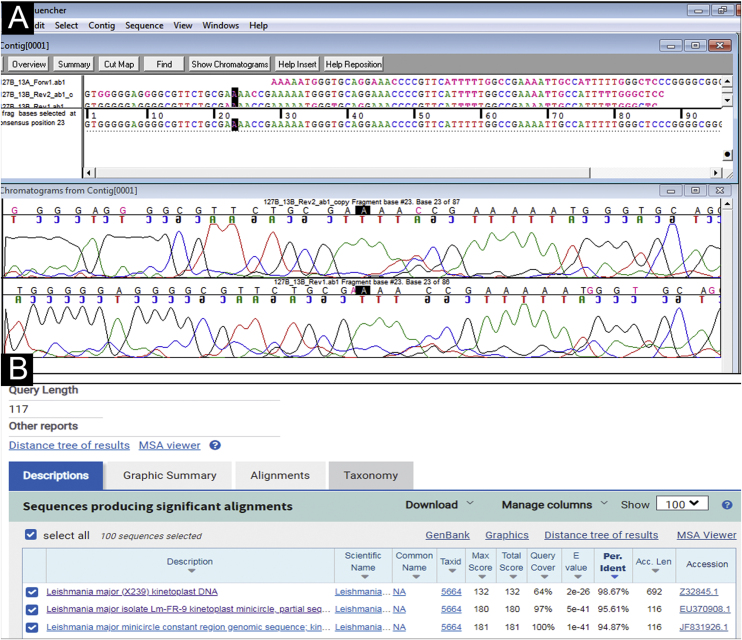
Analysis of DNA sequencing of the PCR product with specific primers for a kinetoplast minicircle DNA region (mitochondrial DNA or kDNA) of *Leishmania spp*. (A) Assembling and editing of the sequences, one sense strand (forward) and two antisense strands (reverse), using the Sequencher™ software and observation of the chromatograms or electropherograms. (B) Comparative analysis performed by aligning the consensus sequence with nucleotide sequences deposited in GenBank-NCBI (BLASTn ‒ blast.ncbi.nih.gov.qBlast.cgi). The sequencing allowed the molecular identification of the *Leishmania major* species complex (BLASTn = Similarity or Maximum Identity: >95%, fragment size: 117 bp).

A 31-year-old Syrian man presented with eleven erythematous-crusted plaques on the limbs, trunk and scalp, in addition to a framed ulcer measuring 1.5 cm, with clear bottom, on the distal region of the right leg (Fig. 1). The lesions had started three months before, without systemic symptoms, after 45 days of having travelled to Homs, Syria. Three family members had similar lesions. Considering the hypothesis of TL, biopsies were performed and histopathology showed a mixed dermal infiltrate and the presence of many amastigote parasites inside macrophages (Fig. 2). However, it was not possible to identify any differences between *L. amazonensis* and *L. major* through the anatomopathological analysis, although the literature reports differences in the size of the parasitophorous vacuoles of these species (larger and with more parasites for *L. amazonensis* and smaller with few parasites for *L. major*).^1^ DNA extraction was performed with the QIAamp™ DNA FFPE tissue kit (QIAGEN) using the fragment from the skin biopsy fixed in formalin and embedded in paraffin, with simple polymerase chain reaction (PCR) and Sanger sequencing being performed for the analysis of the kinetoplast minicircle DNA (kDNA or mitochondrial DNA) of *Leishmania spp*. This genetic target was chosen due to the presence of multiple copies per cell and due to the size of the DNA fragment amplified from the employed primers (kDNA-F, forward: 5’-GTGGGGGAGGGGCGTTCT-3’ and kDNA-R, reverse: 5’-ATTTTACACCAACCCCCAGTT-3’),^2^, ^3^, ^4^ allowing greater sensitivity for parasite detection in the type of analyzed sample. PCR resulted in the specific amplification of a fragment with an approximate size of 116‒120 base pairs (Fig. 3) and DNA sequencing allowed the molecular identification of the *Leishmania major* species complex (Fig. 4), confirming the presence of the non-native agent in the country. Unfortunately, the patient moved and did not return in person, but only via telemedicine, with spontaneous resolution.

## Discussion

In the Middle East, the most often reported etiological agents are *L. tropica* and *L. major*, transmitted by mosquitoes of the genus *Phlebotomus*.^5^ *L. major*, however, does not have a reservoir, being considered an anthroponosis. In Brazil, the most prevalent species are *L. braziliensis*, *L. amazonensis* and *L. guyanensis*, transmitted by mosquitoes of the genus *Lutzomyia*. As for the clinical picture, unlike the classic ulcer with a granulomatous bottom, OWTL is characterized by multiple erythematous papules at the sites of the insect bites, which develop into nodules < 2 cm, ulcerate, and form a crust (“dry ulcers”).^5^ The Montenegro test has a high sensitivity for the detection of a prior *L. major* infection.^6^

In 1991 the cases decreased in Syria, where this disease is endemic, due to the fight against mosquitoes. However, in the late 1990s, an increase was observed, which was attributed to intense rural-urban migration and the deterioration of services, such as garbage collection and basic sanitation.^7^ As of 2011, there have been no more epidemiological data, due to the start of the civil war, which culminated in the destruction of 57% of the public hospitals.^7^ The collapse of health services and mass migration to neighboring countries ensued. Thus, the re-emergence of diseases such as polio, measles, and tuberculosis was observed, in addition to TL, in response to the increase in vectors and the healthcare chaos.

The refugees were settled in border towns, mainly in Turkey, without medical care and living in an environment favorable for the spreading of epidemics. New cases of TL were observed in refugee camps. In Lebanon, there was an increase from six cases between 2000‒2012 to 1,033 cases in 2013 alone, 97% of which were Syrian refugees.^8^

In Brazil, Syrians constitute the predominant group of refugees.^9^ There are situations of vulnerability, political, socioeconomic, language and cultural obstacles, which are barriers to accessing decent health care. Therefore, the diagnosis, treatment, notification and epidemiological analysis are impaired.

Hence, it is possible to discuss the autochthonous transmission of new species of *Leishmania*. In Turkey, non-endemic strains have been detected, even in a patient with no history of travelling, suggesting the introduction of this species.^10^ In the Mediterranean, the competence of the mosquito *Anopheles sergentii* as a vector was verified,^11^ raising the hypothesis of the feasibility of introducing this disease into other European countries. In Brazil, where PCR is not a routinely performed test, there are potential vectors for *L. major*^12^, ^13^ and isolated cases of *L. majo*r-*like* strains, possibly *L. major*,^14^ have been described in the Midwest in the 1970s and 1980s, in patients with no history of travelling.^15^ However, there is lack of information to evaluate the capacity for autochthonous transmission.

## Conclusion

The authors emphasize the importance of this diagnosis in patients from endemic areas, considering the potential for deformities. Moreover, they emphasize the relevance of this report for the epidemiological investigation of the possibility of autochthonous transmission of Old World species in our country.

## Financial support

None declared.

## Authors' contributions

Cindy Tiemi Matsumoto: Data collection, or analysis and interpretation of data; writing of the manuscript or critical review of important intellectual content; intellectual participation in the propaedeutic and/or therapeutic conduct of the studied cases; critical review of the literature; approval of the final version of the manuscript.

Milvia Maria Simões and Silva Enokihara: Intellectual participation in the propaedeutic and/or therapeutic conduct of the studied cases; critical review of the literature.

Marília Marufuji Ogawa: Data collection, or analysis and interpretation of data; effective participation in research orientation; intellectual participation in the propaedeutic and/or therapeutic conduct of the studied cases; critical review of the literature; approval of the final version of the manuscript.

Samira Yarak: Data collection, or analysis and interpretation of data; writing of the manuscript or critical review of important intellectual content; effective participation in research orientation; intellectual participation in the propaedeutic and/or therapeutic conduct of the studied cases; critical review of the literature; approval of the final version of the manuscript.

## Conflicts of interest

None declared.

## References

[1] Real F., Mortara R.A., Rabinovitch M. (2010). Fusion between Leishmania amazonensis and Leishmania major parasitophorous vacuoles: live imaging of coinfected macrophages. PLoS Negl Trop Dis..

[2] Akhoundi M., Downing T., Votýpka J., Kuhls K., Lukeš J., Cannet A. (2017). Leishmania infections: molecular targets and diagnosis. Mol Aspects Med..

[3] Prestes S.R., Guerra J.A., Romero G.A., Magalhaes L.K., Santana R.A., Maciel M.G. (2015). Polymerase chain reaction-based method for the identification of Leishmania (Viannia) braziliensis and Leishmania (Viannia) guyanensis in mucosal tissues conserved in paraffin. Rev Soc Bras Med Trop..

[4] Reale S., Maxia L., Vitale F., Glorioso N.S., Caracappa S., Vesco G. (1999). Detection of Leishmania infantum in dogs by PCR with lymph node aspirates and blood. J Clin Microbiol..

[5] Uzun S., Gürel M.S., Durdu M., Akyol M., Karaman B.F., Aksoy M. (2018). Clinical practice guidelines for the diagnosis and treatment of cutaneous leishmaniasis in Turkey. Int J Dermatol..

[6] Alvarado R., Enk C., Jaber K., Schnur L., Frankenburg S. (1989). Delayed-type hypersensitivity, and lymphocyte proliferation in response to Leishmania major infection in a group of children in Jericho. Trans R Soc Trop Med Hyg..

[7] Al-Salem W.S., Pigott D.M., Subramaniam K., Haines L.R., Kelly-Hope L., Molyneux D.H. (2016). Cutaneous Leishmaniasis and Conflict in Syria. Emerg Infect Dis..

[8] Saroufim M., Charafeddine K., Issa G., Khalifeh H., Habib R.H., Berry A. (2014). Ongoing epidemic of cutaneous leishmaniasis among Syrian refugees, Lebanon. Emerg Infect Dis..

[9] gov.br [Internet]. Coordenação-Geral do Comitê Nacional para os Refugiados. Refúgio em números, 4ª edição. Ministério da justiça e segurança pública. 2019. Available from: https://www.gov.br/mj/pt-br/assuntos/seus-direitos/refugio/refugio-em-numeros-e-publicacoes/anexos/refugio_em_numeros-4e.pdf.

[10] Özbilgin A., Gencoglan G., Tunali V., Çavuş I., Yıldırım A., Gündüz C. (2020). Refugees at the Crossroads of Continents: A Molecular Approach for Cutaneous Leishmaniasis Among Refugees in Turkey. Acta Parasitol..

[11] Khamesipour A., Rathb B. (2016). Refugee health and the risk of cutaneous Leishmaniasis in Europe. Int J Infect Dis..

[12] Guimarães A.C., Nogueira P.M., Silva S.d.O., Sadlova J., Pruzinova K., Hlavacova J. (2018). Lower galactosylation levels of the Lipophosphoglycan from Leishmania (Leishmania) major-like strains affect interaction with Phlebotomus papatasi and Lutzomyia longipalpis. Mem Inst Oswaldo Cruz..

[13] Soares R.P.P., Turco S.J. (2003). Lutzomyia longipalpis (Diptera: Psychodidae: Phlebotominae): a review. An Acad Bras Ciênc..

[14] Almeida L.V., Luís Reis-Cunha J.L., Coqueiro-Dos-Santos A., Rodrigues-Luís G.F., Baptista R.P., de Silva S.O. (2021). Comparative genomics of Leishmania isolates from Brazil confirms the presence of Leishmania major in the Americas. Int J Parasitol..

[15] Silva S.O., Wu A.A., Evans D.A., Vieira L.Q., Melo M.N. (2009). Leishmania sp. isolated from human cases of cutaneous leishmaniasis in Brazil characterized as Leishmania major-like. Acta Trop..

